# Health resource mobilization: state or government policies?

**DOI:** 10.1590/acb409025

**Published:** 2026-01-30

**Authors:** Everson Artifon, Marcio Roberto Facanali, José Pinhata Otoch

**Affiliations:** 1 Universidade de São Paulo – Faculdade de Medicina – Departamento de Cirurgia – São Paulo (SP) – Brazil.; 2 Universidade de São Paulo – Faculdade de Medicina – Hospital das Clínicas – Departamento de Gastroenterologia – São Paulo (SP) – Brazil.

Health resource mobilization plays a pivotal role in sustaining the four fundamental pillars of healthcare: innovation, research, education, and patient care. It represents a complementary source of funding that addresses multiple demands which cannot be covered exclusively by regular government budgets. However, the seasonal nature of political interests often shifts structural, long-term projects into the background, in favor of short-term, media-driven initiatives designed to reinforce political bases. It is worth recalling that, in Brazil, 50% of parliamentary amendments must be mandatorily directed to the health sector.

The pool of health resources encompasses several initiatives, particularly parliamentary amendments at the federal, state, and municipal levels, as well as funding from governmental agencies such as the Financier of Studies and Projects (FINEP), State Research Foundations, Coordination for the Improvement of Higher Education Personnel (CAPES), Department of Science and Technology of the National Health Fund, and the National Council for Scientific and Technological Development (CNPq). In this scenario, it is crucial that public agents and institutional leaders engaged in governmental relations focus on projects aligned with public health policies that are sustainable, structured, and capable of transcending political cycles. Previous experiences have already highlighted fundraising as a tool that extends beyond financing, serving as an instrument for institutional development and long-term professional growth^1^.

The key distinction lies between government policies and state policies. While government policies are subject to discontinuation, reflecting the ideologies and priorities of the group in power, state policies are institutionalized, long-lasting, and capable of remaining effective across successive administrations. When a public policy is developed with broad social support, robust planning, and sustainable financing, it evolves into a state policy^2^.

Thus, institutional relations must guide legislators to channel health-related amendments toward actions that consolidate the constitutional right to health. This approach converts short-term political strategies into sustainable state policies, ensuring continuity and stability. The international literature reinforces that sustainability in health cannot rely on temporary solutions but must be institutionalized as part of an enduring governance structure^3^^,^ 4.

Finally, fostering a culture of resource mobilization aligned with state-level health policies is essential. Given the need to strengthen the fundamental pillars of healthcare, such alignment represents not only a strategic organizational shift but also a commitment to long-term public health sustainability.

## Data Availability

All data are presented in the article.
